# Can the Complexity of Diagnosing Pulmonary Embolism in Patients With Obesity and Multiple Comorbidities Provide an Explanation of the Obesity Paradox?

**DOI:** 10.7759/cureus.94246

**Published:** 2025-10-09

**Authors:** Martin V Balzan, Alison Galea

**Affiliations:** 1 Respiratory Medicine, Mater Dei Hospital, Msida, MLT; 2 General Medicine, Mater Dei Hospital, Msida, MLT

**Keywords:** all-cause mortality, delayed diagnosis, misdiagnosis, obesity paradox, pulmonary embolism

## Abstract

This report describes three cases of patients with obesity who had chronic comorbidities and presented with documented causes of dyspnea before hospital admission. The challenges in recognising and diagnosing pulmonary embolism (PE) in these patients are highlighted and discussed in light of current literature. It is possible that delayed or missed diagnoses are common among patients with a history of dyspnea, a symptom frequently observed in individuals with obesity. A hypothesis is proposed based on existing medical literature to explain the so-called "obesity paradox." This hypothesis suggests that the seemingly lower relative risk of mortality from PE in patients with obesity, compared to their counterparts without obesity, may stem from a higher rate of missed diagnoses in the former who have multiple comorbidities, many of which often present with dyspnea, besides other symptoms. Such a situation could lead to misleading data indicating better outcomes due to selection bias, where healthier patients with obesity but without comorbid conditions are more easily identified and receive prompt treatment. In contrast, delays or missed diagnoses in more vulnerable patients with obesity who have multiple health issues may result in significantly worse outcomes, potentially explaining the increased all-cause mortality associated with obesity. While this paper specifically examines PE, the same principle may apply to other medical emergencies involving patients with obesity who present with acute dyspnea. Specific studies are needed to simultaneously assess the outcomes for both diagnosed and undiagnosed patients with obesity to prove or disprove this hypothesis.

## Introduction

Obesity is a significant risk factor for various comorbidities, which can be classified into three categories: metabolic, anatomical, and psychological. [1] A meta-analysis of 239 prospective studies found that the risk of all-cause mortality increases with higher levels of obesity. Specifically, the hazard ratio (HR) for obesity grade 1 (body mass index (BMI) 30.0-<35.0 kg/m²) was 1.45 (95% CI 1.41-1.48). For obesity grade 2 (BMI 35.0-<40.0 kg/m²), the HR was 1.94 (95% CI 1.87-2.01), and for obesity grade 3 (BMI 40.0-<60.0 kg/m²), the HR rose to 2.76 (95% CI 2.60-2.92) [2]. The metabolic consequences of obesity include type 2 diabetes, metabolic dysfunction-associated steatosis, liver disease, hypercholesterolemia, chronic kidney disease, and atherosclerotic cardiovascular disease [1].

Banack et al. have challenged the conventional view by suggesting that, in certain clinical populations such as individuals with cardiovascular disease, cancer, and respiratory or renal disorders, obesity may actually provide a survival advantage during acute illness [3]. Similar findings were reported by Shachar et al. in relation to pulmonary embolism (PE), showing an odds ratio of 0.5 for in-hospital mortality [4]. This is commonly referred to as the “obesity paradox.”

We present three complex cases in which PE was diagnosed in patients with a BMI greater than 30 kg/m², who also had multiple underlying health conditions. These co-morbidities contributed to varying degrees of dyspnea at the time of presentation. These cases illustrate how the presence of various causes of acute or chronic dyspnea in patients with obesity, combined with the often subtle symptoms of PE, can result in a delayed or missed diagnosis.

We propose a hypothesis that the high rate of missed diagnoses in patients with obesity who have multiple co-morbidities may lead to overly optimistic survival data in diagnosed and treated patients, potentially explaining the obesity paradox.

## Case presentation

Case 1

A 54-year-old female, a non-smoker, with a BMI of 35 kg/m^2^, was known to suffer from bronchial asthma on budesonide 200 µg, two puffs BD (*bis in die*; twice a day), and formoterol aerolizer 12 mcg once daily. Further medical history included hypertension, ischemic heart disease, and depression. She was on bendrofluazide 5 mg daily, valsartan 80 mg BD, and paroxetine 20 mg BD.

In 2012, the patient presented to the emergency department with a three-week history of persistent exertional dyspnea that did not improve with the use of short-acting beta agonists, methacholine antagonists via nebuliser, or a full course of antibiotics (co-amoxiclav). She also mentioned experiencing occasional pain in her left calf. Upon auscultation, there was no wheezing detected in her chest; however, her oxygen saturation was 90% on room air, and her heart rate was 107 beats per minute. Her blood pressure was stable at 118/67 mmHg, and her temperature was 36.8°C.

**Figure 1:**
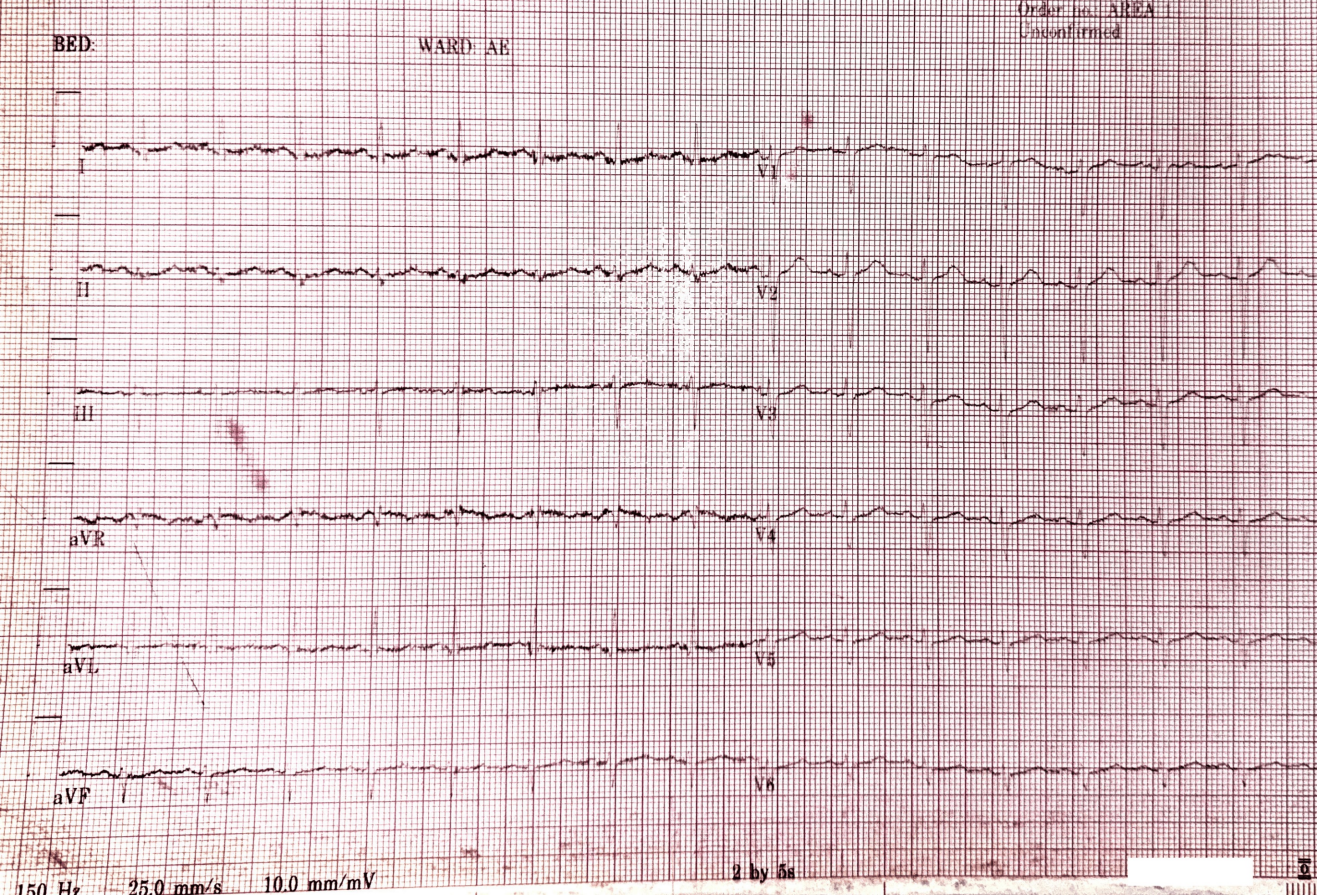
Electrocardiogram on admission (Case 1).

A full blood count, renal profile, and random blood glucose were unremarkable. Inflammatory markers, including C-reactive protein (CRP) and erythrocyte sedimentation rate (ESR), were slightly elevated. Qualitative D-dimer was positive (Table 1). Arterial blood gases (ABGs) on room air showed respiratory alkalosis (Table 2). Electrocardiography (ECG) showed sinus tachycardia, and the chest X-ray (CXR) was normal (Figure 1).

**Table 1:** Blood test results of the three cases D-Dimer in Case 1 was qualitative, in Case 2 it was quantitative, and in Case 3 it was not performed (pulmonary embolism discovered incidentally) Renal impairment was noted in Case 2; CRP was raised in Case 2 and grossly raised in Case 3 because of sepsis; Case 3 had neutropenia secondary to sepsis; haemoglobin was low in cases 2 and 3, and in Case 3, aPTT was raised secondary to DIC. DIC: diffuse intravascular coagulation; aPTT: activated partial thromboplastin time; INR: international normalised ratio; PT: prothrombin time

Parameters	Patient Values in Case 1	Patient Values in Case 2	Patient Values in Case 3	Normal Range
D-Dimer	Positive	2673	n/a	0-500 ng/ml
Urea	6.70	9.80	12.90	1.7-8.3 mmol/L
Creatinine	87	141	89	53-88 umol/L
Sodium	137	145	136	136-145 mmol/L
Potassium	4.12	3.79	3.47	3.5-5.1 mmol/L
CRP	17	31.2	314	0-10 mg/l
White Blood Cells	9.50	10.72	0.29	3.5-11 x 10^9^/L
Neutrophils	5.73	7.32	0.06	2.5-7.5 x 10^9^/L
Lymphocytes	2.81	2.04	0.19	1.6-3.5 x 10^9^/L
Monocytes	0.70	0.87	0.02	0.2-0.8 x 10^9^/L
Eosinophils	0.25	0.42	0.00	0-0.4 x 10^9^/L
Basophils	0.03	0.04	0.00	0-0.1 x 10^9^/L
Haematocrit	42.9	32.7	28.7	36-48%
Mean Cell Volume	86.5	95.7	85.2	76-95 fL
Mean Cell Haemoglobin	30.0	28.9	28.8	27-34 pg
Mean Cell Haemoglobin Concentration	34.7	30.2	33.8	30-36 g/dL
Red Cell Distribution Width	14.0	16.5	14.2	12.3-14.9%
Mean Platelet Volume	10.3	10.0	10.7	6-12 fL
Reticulocytes Abs	93.7	132.7	4.20	20-130 x 10^9^/L
Haemoglobin	14.9	9.9	9.7	11.5-16.5 g/dL
Platelets	342	243	69	140-400 x 10^9^/L
Red Cell Count	5.00	3.42	3.37	3.9-5.6 x 10^12^/L
INR	1.02	1.00	1.18	0.94-1.06 ratio
PT	10.80	10.80	12.60	9.96-11.24 sec
aPTT	25.4	22.5	67	19.26-25.59 sec

**Table 2:** Arterial blood gases in the three cases All three cases were hypoxaemic. Cases 1 and 3 showed a type I (restrictive) respiratory deficit, while Case 2 showed a partial pressure of CO2 (pCO_2_) in the plasma at the upper limit of normal, suggesting a ventilation deficit. The raised bicarbonate  (HCO3) level indicates metabolic compensation. sO_2_: oxygen saturation; pO_2_: oxygen partial pressure

Parameters	Case 1 Values	Case 2 Values	Case 3 Values	Normal Range
pH	7.48	7.44	7.36	7.35 - 7.45
pCO_2_	31	44	38	35.0 - 45.0 mmHg
pO_2_	73	54	61	80.0 – 100 mmHg
sO_2_	95	93	92	95.0 - 98.0 %
HCO_3_	25	31	23	22.0 – 26.0 mmol/L

**Figure 2:**
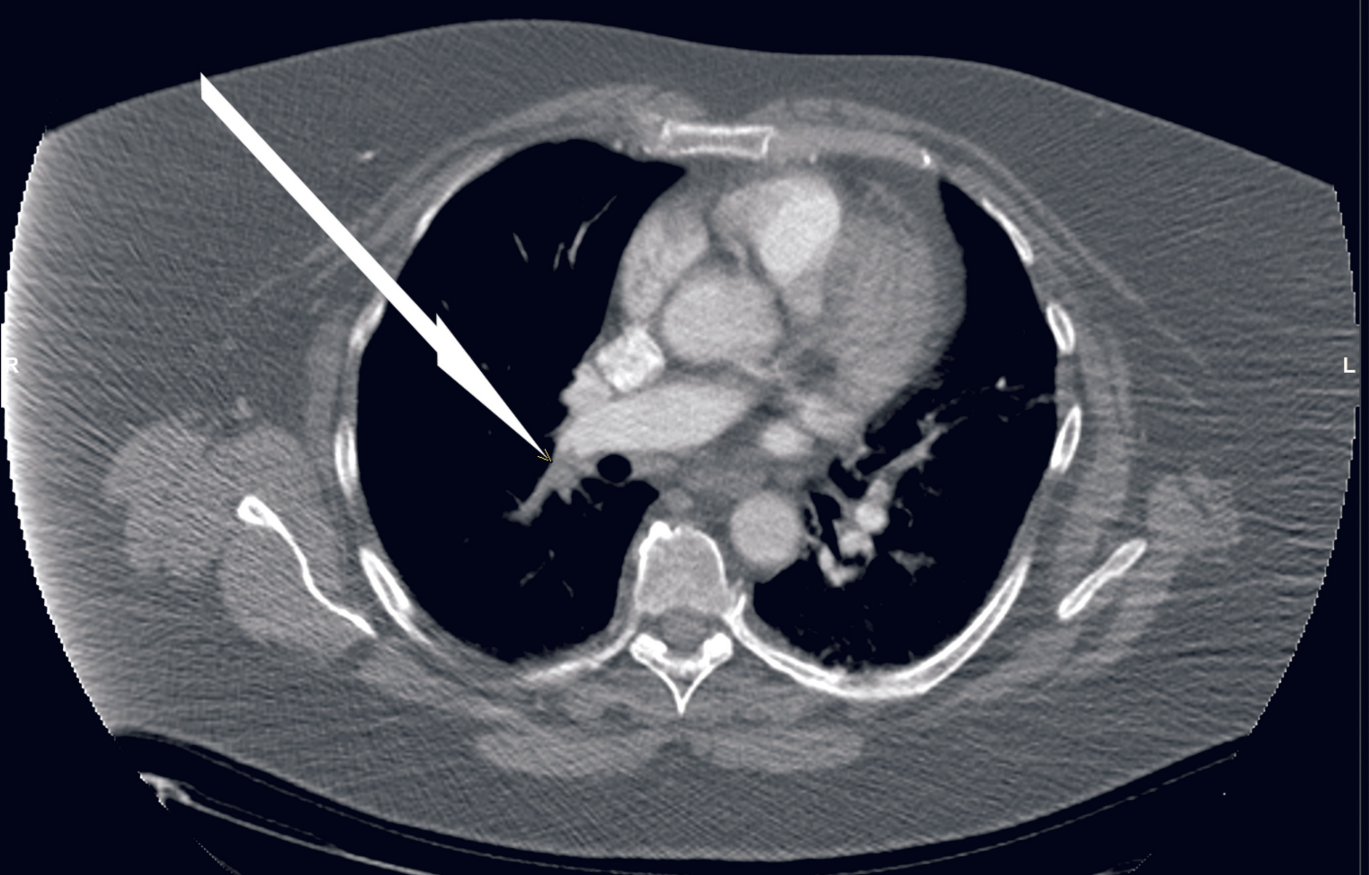
CT pulmonary angiogram, axial view (Case 1). Arrow indicates the grey area without dye in the right pulmonary artery, which is the embolus.

A computed tomography pulmonary angiogram (CTPA) confirmed a diagnosis of a PE with a thrombus in the right lower lobe arteries and several small thrombi in the segmental and subsegmental branches of the pulmonary artery (Figure 2).

A transthoracic echocardiogram (TTE) was not performed. The patient was admitted for cardiac monitoring and supplemental oxygen therapy and was started on enoxaparin at a dose of 1.5 mg/kg daily. Over the next few days, her dyspnea improved, and she was discharged on oral warfarin adjusted according to international normalised ratio (INR). A TTE conducted a year later was reported as normal.

The patient experienced one episode of SARS-CoV-2 infection in 2021 and had four additional admissions for asthma since 2012. CTPA was repeated twice in 2013 and once in 2020 due to episodes of dyspnea, and all three scans were reported as normal. A TTE in 2022 showed normal left ventricular size, with an ejection fraction of 55%. However, the right ventricle was mildly dilated, with mild tricuspid regurgitation. By 2025, the patient was still alive.

Case 2

A 72-year-old male with a BMI of 43 kg/m², an ex-smoker with a 20-pack-year history, and a known case of chronic obstructive pulmonary disease (COPD), obstructive sleep apnea, and obesity hypoventilation syndrome (OHS). This condition had been confirmed by a sleep study, which indicated an Apnea Hypopnea Index (AHI) of 42.3 with a resting pCO2 of 43 mmHg. 

His additional medical history included hypertension and peripheral vascular disease, which required left superficial femoral artery percutaneous angioplasty in 2021. Four years before this presentation, he had been diagnosed with prostate adenocarcinoma, which was treated conservatively with radiotherapy; however, he was not on any specific medication at the time of admission. His lung function tests revealed a forced expiratory volume in one second (FEV1) of 1.88L (69%), forced vital capacity (FVC) of 2.83L (79%), and an FEV1/FVC ratio of 66.4% (GOLD Stage 2 Moderate COPD). His medications included umeclidinium 62.5 mcg one puff daily, salmeterol 100 mcg two puffs BD, aspirin 75 mg daily, and valsartan 80 mg daily. 

He had two previous admissions for acute dyspnoea; however, the CTPA performed in 2013 and 2020 had been reported as normal. In 2024, he presented to the emergency department with a three-day history of central compressive chest pain that was non-radiating and non-pleuritic, accompanied by exertional dyspnea, and occasionally associated with vaguely described left calf pain. At the time of presentation, he was recovering from a recent upper respiratory tract infection. The patient was hemodynamically stable, with a blood pressure of 105/60 mmHg and a pulse of 95 beats per minute (bpm). 

**Figure 3:**
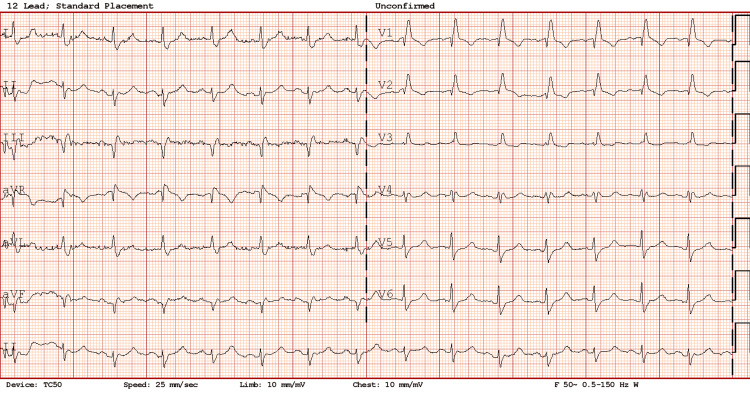
Electrocardiogram on admission (Case 2)

Upon physical examination, the chest appeared unremarkable, and there were no clinical signs of deep vein thrombosis (DVT). ABG analysis showed a partial pressure of carbon dioxide in the plasma at the upper limit of normal, suggesting a ventilation deficit. The raised bicarbonate level indicated metabolic compensation (Table 2). A D-dimer test was ordered due to the left calf pain associated with chest pain, which returned elevated at 2675 ng/ml. Other blood tests indicated mild renal impairment and normochromic normocytic anaemia (Table 1). An ECG revealed a right bundle branch block, which was noted to have been previously present (Figure 3).

**Figure 4:**
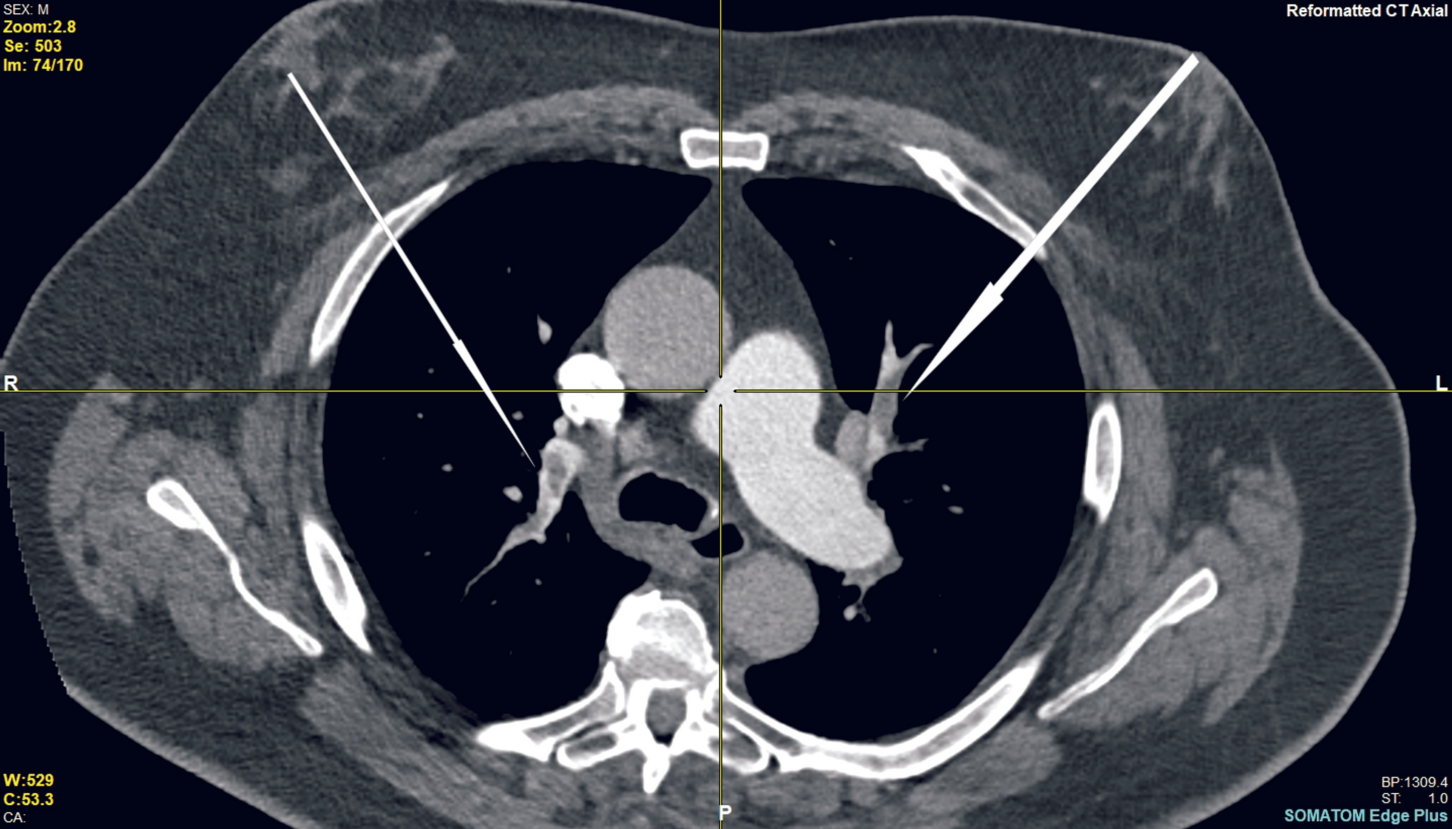
CT pulmonary angiogram, axial view (Case 2). Arrows indicate bilateral pulmonary emboli in right and left pulmonary arteries.

**Figure 5:**
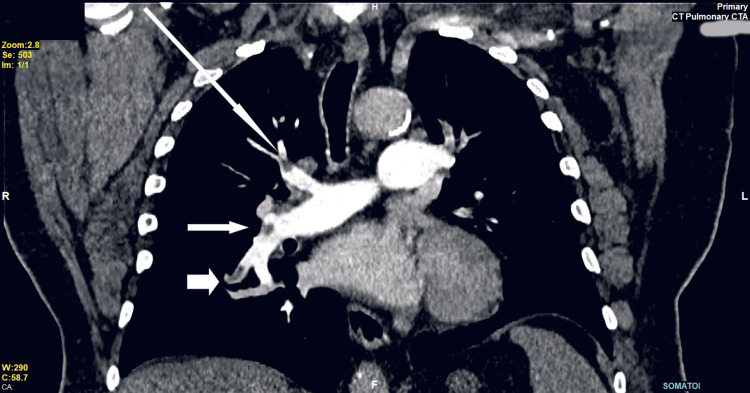
CT pulmonary angiogram, coronal view (Case 2). Arrows indicate pulmonary emboli.

A CXR was unremarkable except for minor atelectatic changes in the left lower lung zone, which were noted in past imaging. A CTPA showed multiple filling defects in the segmental pulmonary arteries bilaterally (Figures 4, 5).

The patient was loaded on enoxaparin at 1.5 mg/kg daily and admitted to a monitor bed. The admission was uneventful, and he was discharged after four days on rivaroxaban 20 mg daily.

A TTE performed seven months after admission showed that the left ventricle was not dilated and had good systolic function, with no obvious regional wall motion abnormalities. A septal bounce was noted, possibly secondary to a bundle branch block. Diastolic function was normal. The right ventricle was also not dilated and contracted well. There was no evidence of tricuspid valve regurgitation. Estimated pulmonary artery pressure was not elevated, and the inferior vena cava (IVC) was not dilated.

Case 3

The patient was a 69-year-old woman with a BMI of 33kg/m^2^, a non-smoker, a known case of insulin-dependent diabetes mellitus (IDDM), neurosarcoidosis with minor chest involvement, with a history of ST-elevation myocardial infarction, heart failure with reduced ejection fraction (HFrEF) on echocardiography, hyperlipidemia and OSA but was not compliant to continuous positive airway pressure (CPAP) machine. Her treatment included metformin 1 g BD, aspirin 75 mg daily, clopidogrel 75 mg daily, venlafaxine 75 mg BD, enalapril 5 mg BD, insulin glargine 24 units subcutaneous daily, methotrexate 20 mg weekly, folic acid 5 mg daily, rosuvastatin 10 mg at night, ezetimibe 5 mg at night, and carvedilol 3.125 mg BD.

She presented to the emergency department in view of chest pain, diaphoresis, and dyspnea. The patient was hemodynamically stable on presentation with a blood pressure of 121/64 mmHg, pulse of 83 bpm, and oxygen saturation (SpO2) 100% on room air. She was afebrile. Clinical examination was unremarkable.

She was treated for a non-ST-elevation myocardial infarction (NSTEMI) with an elective percutaneous coronary intervention (PCI) x3 to the left main coronary artery, left anterior descending, and left circumflex arteries. TTE revealed a normal right ventricular size but with impaired function, with a dilated left ventricle (LV) and moderate concentric LV hypertrophy. Severely impaired LV function with an ejection fraction of 33% using Simpson’s biplane method was noted, with evidence of impaired relaxation. Aortic sclerosis was evident with a severely calcified mitral valve but no evidence of mitral stenosis.

**Figure 6:**
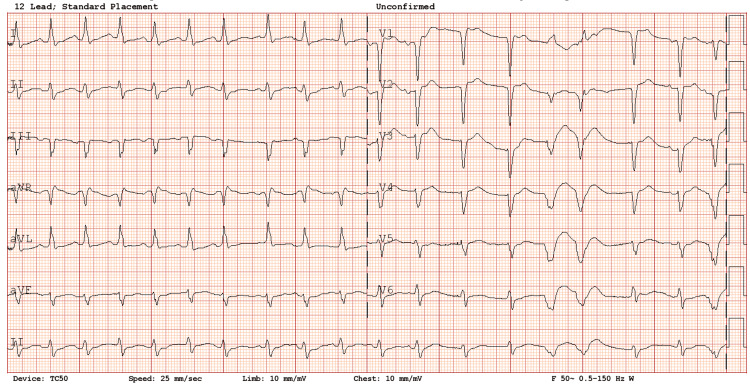
ECG before admission to intensive care shows q waves of previous myocardial infarction (V1-V3) (Case 3).

On recovery, the patient was noted to be dyspneic with an SpO2 of 91% on room air. Heart rate was 101 bpm, BP 114/80 mmHg. ABG on room air showed type I respiratory failure (Table 2). Her electrocardiogram showed Q waves in the V1 to V3 (Figure 6).

Right fore-arm cellulitis was noted at the venous cannulation site. Blood tests were indicative of acute sepsis with neutropenia, thrombocytopenia, and diffuse intravascular coagulation (Table 1). Chest X-ray showed clear lung fields.

The patient was transferred to intensive care with a provisional diagnosis of sepsis, and started on high-dose intravenous teicoplanin, aztreonam, and metronidazole antibiotics, and methotrexate was stopped to reduce immunosuppression. 

**Figure 7:**
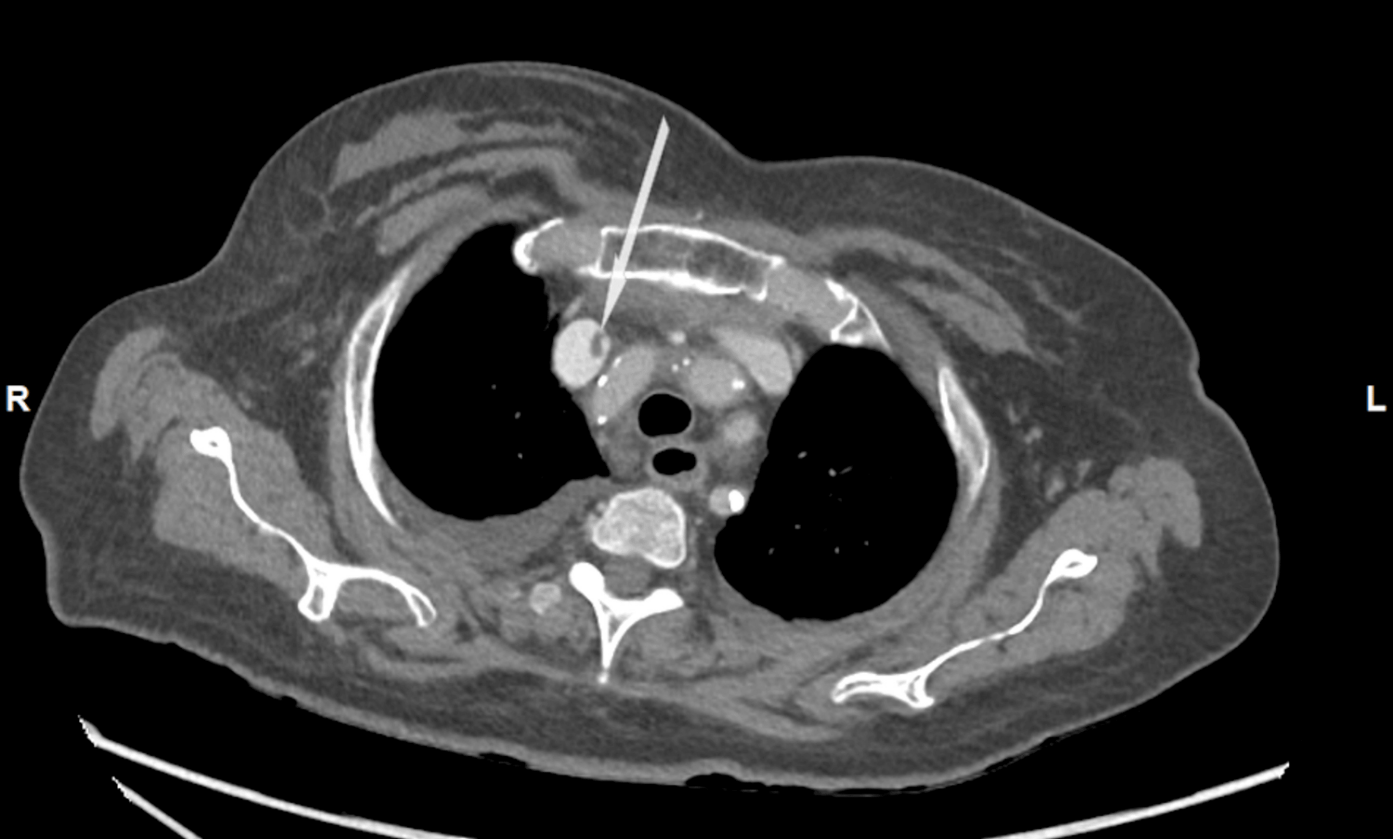
CT thorax, abdomen and pelvis, axial view (Case 3). Arrow shows thrombus in the superior vena cava. Bilateral small pleural effusions present.

**Figure 8:**
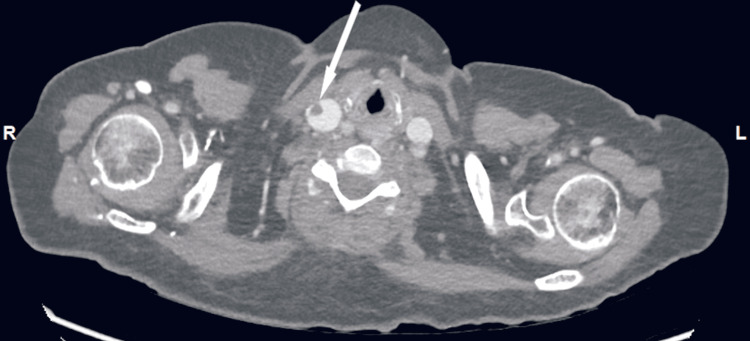
CT thorax, abdomen and pelvis, axial view (Case 3). Arrow shows thrombus extending into the right jugular vein.

A CT thorax, abdomen, and pelvis (TAP) was done in an effort to establish an alternative source of the sepsis by the caring physicians. Non-occlusive linear filling defects were noted in the superior vena cava (Figure 7) extending to the right internal jugular (Figure 8), reflecting intravascular thrombus.

**Figure 9:**
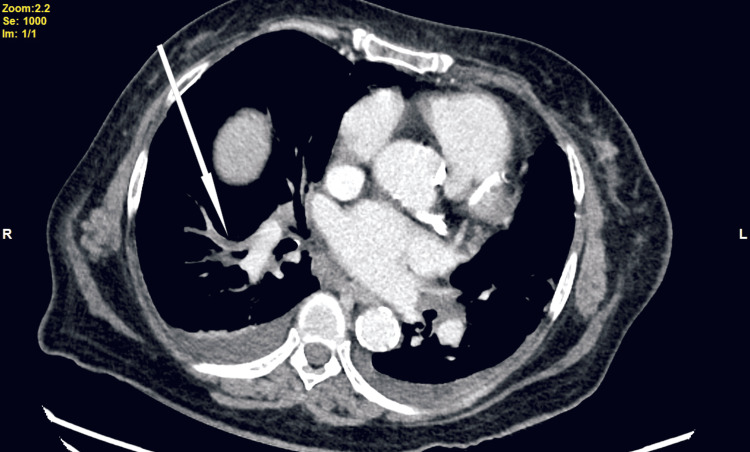
CT thorax, abdomen and pelvis, axial view (Case 3). Arrow indicates grey area of thrombus occluding a branch of the right pulmonary artery. (Small bilateral pleural effusions present.)

Furthermore, on cross-sectional imaging (Figure 9), there were multiple extensive filling defects within the segmental and sub-segmental pulmonary arteries of the right lower lung lobe in keeping with pulmonary embolism. In view of the multiple thromboses, she was started on subcutaneous enoxaparin 1.5 mg/kg daily. Bilateral pleural effusion was noted.

The patient gradually improved after cardiac rehabilitation and was discharged, and rivaroxaban 15 mg BD was added to her previous treatment regimen. CTPA examinations performed two months and one year later, following further episodes of dyspnea, were both reported as normal. The main pulmonary arteries were patent and free from thrombus.

## Discussion

The simplified Geneva Score [5] presented in Table 3 indicated that three cases had scores of ≥3, suggesting an intermediate probability of PE. In contrast, the Wells score [6] shown in Table 4 classified all three cases as unlikely for PE, with scores of ≤4, indicating a low probability in the two-tier model.

**Table 3:** Simplified Revised Geneva score the three cases This table summarises the clinical findings needed to tally the Simplified Revised Geneva score [5]. DVT: deep vein thrombosis; PE: pulmonary embolism

Predictive Variable	Simplified Revised Geneva Score	Case 1	Case 2	Case 3
Previous PE or DVT	1	0	0	0
Heart rate 75-94 bpm	1	0	0	0
Heart rate >95 bpm	2	2	2	2
Surgery or fracture of the lower limbs within one month	1	0	0	0
Active malignancy (or considered cure <1 year)	1	0	0	0
Hemoptysis	1	0	0	0
Unilateral lower limb pain	1	1	1	1
Tenderness on lower limb deep venous palpation and unilateral oedema	1	0	0	0
Age 65 years or over	1	0	1	1
Total		3	4	4

**Table 4:** Clinical features and Wells score for the three cases This table summarises the clinical findings needed to tally the Wells score [6] for cases 1 to 3. DVT: deep vein thrombosis; PE: pulmonary embolism

Predictive Variable	Wells Score	Case 1	Case 2	Case 3
Clinical signs of DVT	3	0	0	0
Alternative diagnosis less likely than PE	3	0	0	0
Heart rate >100 bpm	1.5	1.5	0	1.5
Immobilization (>3 days) or surgery within 4 weeks	1.5	0	0	1.5
Previous DVT/PE	1.5	0	0	0
Hemoptysis	1	0	0	0
Malignancy (recent or palliative)	1	0	0	0
Total		1.5	0	3

Case 1

The patient suffered from bronchial asthma, and initially, her family physician treated her worsening dyspnea empirically as a case of asthma with nebulised beta agonists and antibiotics. Although wheezing was absent, her ABG test indicated a type I respiratory deficit. A CXR showed borderline cardiomegaly, but her echocardiogram was normal, and there were no signs of acute pulmonary oedema. Calf pain was only a minor complaint for the patient; however, a positive D-dimer test raised suspicion of pulmonary embolism. A CTPA revealed multiple obstructions in the right pulmonary arteries, suggesting that several episodes of thrombosis may have occurred over a significant period without being diagnosed.

Case 2

The second patient had documented sleep apnea, along with obesity hypoventilation syndrome. He had a significant smoking history, and lung function tests indicated moderate COPD. Additionally, he had a history of prostatic cancer, which had been treated a few years prior and was now stable, with no active treatment. This combination led to type II (ventilatory) failure, which is typically not associated with PE. The patient's worsening dyspnea was initially attributed to a possible lower respiratory tract infection. Although there were no clinical signs of DVT, the combination of calf pain and chest pain alerted the clinicians to check the D-dimer levels and eventually proceed to a CTPA. As multiple emboli were identified, this condition had likely developed over a considerable period, resulting in the patient's dyspnea that ultimately necessitated hospitalisation.

Case 3

This patient, in addition to her obesity, had multiple comorbidities, including OSA, diabetes mellitus, hyperlipidemia, a previous history of acute myocardial infarction, and chronic low ejection fraction LV failure, alongside chronic neurosarcoidosis currently treated with methotrexate, an immunosuppressant. During her current hospitalisation, she was treated for an acute myocardial infarction. It is possible that during her initial recovery, she developed a hospital-acquired infection at the cannula site in her arm, leading to sepsis and diffuse intravascular coagulation. The patient experienced type I respiratory failure and was hemodynamically unstable. A normal CXR made acute LV failure unlikely. There was an urgent need for high-dose intravenous antibiotic therapy and transfer to intensive care for supportive treatment. The CT scan revealed thrombi in the vena cava and the internal jugular vein, strongly supporting the diagnosis of intravascular coagulation along with multiple pulmonary emboli. Despite her obesity and the associated comorbidities, this patient improved and was discharged, highlighting her resilience in the face of such a severe illness.

It is important to note that cases 1 and 3 had three and two admissions, respectively, after their first episode of PE, all five due to acute dyspnea, which required CTPA. However, the results had been negative in all five instances. In Case 2, there had been two previous admissions with dyspnoea requiring CTPA, which were also reported as normal. These episodes were not caused by thrombosis but were related to other co-morbidities. Specific details of these seven episodes have not been included in this report so as to maintain focus on the thrombotic episodes; however, these events highlight the challenges of the differential diagnosis in such patients once the CT is not conclusive. Patients with these conditions are at higher risk from exposure to both intravenous dye and radiation, despite the importance of establishing the correct diagnosis.

How can the obesity paradox be possibly explained?

These three complex cases exemplify how obese patients with varying causes of acute or worsening chronic dyspnea can complicate the recognition and diagnosis of pulmonary embolism at the time of presentation. 

Dyspnea associated with multiple comorbidities appears to be more prevalent among patients with obesity. In a study conducted by Goh et al., which included 4.6 million individuals, it was found that elderly patients (ages 65-75 years) and middle-aged patients with obesity (ages 45-55 years), as well as younger adults (ages 25-35), were more likely to report experiencing dyspnea compared to their counterparts without obesity. The odds ratios (ORs) for these groups were 3.64, 3.2, and 2.76, respectively. The study encompassed patients with a variety of conditions, including asthma, heart failure, chronic ischemic heart disease, primary hypertension, diabetes mellitus, metabolic syndrome, other chronic obstructive pulmonary diseases, various lung disorders, interstitial pulmonary diseases, congenital malformations of the respiratory system, PE, sleep apnea, and vaping-related disorders [7].

Obese patients seem to have an increased risk of developing venous thromboembolism (VTE). A study by Frischmuth et al. examined 36,341 patients in Norway from 1996 to 2020 and found that the HR for VTE was 1.86 (95% confidence Interval (CI): 1.58-2.2) for patients with obesity and 1.4 (95%CI: 1.21-1.61) for patients who were overweight [8]. Additionally, Gregson et al. reported an HR of 1.43 (95%CI: 1.33-1.50) for a higher BMI and 1.52 (95%CI: 1.41-1.65) for an elevated waist-to-hip ratio related to VTE in a dataset of 1.13 million patients [9].

Garg et al. conducted a study involving 1.9 million individuals aged over 18 years who were admitted to hospitals in the United States with PE between 2016 and 2020. They reported that patients with obesity experienced better outcomes than patients without obesity, with an OR for mortality of 0.63 (95%CI, 0.60-0.65). This phenomenon is often referred to as the "obesity paradox" in the context of acute PE [10].

Similarly, a study by Keller et al. involving more than 345,000 patients with acute PE in Germany found a reduced OR for in-hospital mortality of 0.74 (95%CI, 0.71-0.77; P < .001), independent of factors such as age, sex, comorbidities, and reperfusion therapies [11]. Additionally, a study by Alkhalfan et al. reported a significantly lower 30-day risk of mortality from thromboembolism for patients with obesity, with an HR of 0.25 among 248 patients examined between 2020 and 2023 [12].

Kwok et al. reviewed a total of 18 studies, which included 2,053 patients with a diagnosis of PE, reported delayed or missed diagnosis of PE in 53.7% of hospital inpatients, and 37.9% of patients who died in intensive care who had undergone autopsy [13]. This alarming number of missed diagnoses suggests that the odds for survival data of patients admitted with a diagnosis of PE may be considered as optimistically biased or, at best, incomplete. 

Alonso-Martinez et al. reported a misdiagnosis of 50% of patients and that higher age, more days of delay, and the absence of syncope or sudden onset dyspnea were factors associated with misdiagnosis [14]. Furthermore, the authors investigated which concurrent diagnoses were most common in patients with wrong or delayed diagnosis in acute pulmonary embolism. All were diseases causing some degree of coincidental respiratory discomfort, namely heart failure (31%), bronchitis (35%), pneumonia (13%), and COPD (15%). The average Wells score was 3 [14]. However, unfortunately, body weight was not evaluated in this study.

Patients with obesity often experience multiple coexisting diseases that can lead to acute dyspnea, which may explain why the diagnosis of PE is frequently delayed or missed. In a review conducted by Van Maanen et al., analysing 12 studies, the average diagnostic delay for PE was found to be 6.3 days (95%CI: 2.5-15.8). The authors identified that certain factors, such as coughing, chronic lung disease, and heart failure, were associated with longer diagnostic delays. In contrast, symptoms like chest pain, recent surgery, and hypotension were linked to a quicker diagnosis [15]. Although the review did not specifically mention obesity, it is important to note that many conditions contributing to delays in diagnosis result in dyspnea, a symptom commonly experienced by patients with obesity [7].

Smith et al. conducted a study on 400 patients with morbid obesity, revealing an average BMI of 29.5 (interquartile range: 25.4-35.1) among those diagnosed using CTPA. The study found that 18.3% of these patients experienced a delayed diagnosis of more than 12 hours. The OR for delayed diagnosis was 1.7 (CI: 1.0 - 2.8, p < 0.05). Furthermore, treatment with heparin was delayed by an average of 3.6 hours compared to 2.5 hours in patients without obesity [16]. More specifically, Smith et al. in another study reported that patients over 65 years of age, who also had morbid obesity and cardiovascular disease, were diagnosed with acute PE later than those who had recent immobility and tachycardia [17].

Hawley et al. identified several factors that hinder clinicians’ ability to accurately diagnose PE in patients with obesity. During history taking, evaluating dyspnea becomes challenging due to the chronic nature of shortness of breath. The physical examination is complicated by a thick layer of adipose tissue, which limits effective auscultation. Additionally, ankle swelling accompanied by chronic skin changes is common among these patients [18].

Baseline measurements for physical signs, such as tachycardia and respiratory rate, may be elevated, while ankle oedema may be a chronic condition. Furthermore, hypoxemia can also be chronic, and levels of D-dimer and brain natriuretic peptide (BNP), as well as pulmonary hypertension, may be persistently elevated [18].

In patients with obesity, a weight-adjusted contrast dose may be necessary when using an increased administration rate for CTPA protocols. This adjustment can significantly enhance pulmonary artery (PA) visibility in these patients, leading to higher-quality scans and a reduction in the number of nondiagnostic and indeterminate CTPA examinations [18]. It is reasonable to conclude that if contrast doses are not adjusted for a patient's weight, the likelihood of obtaining false-negative results from CTPA increases.

The joint European Society of Cardiology (ESC) and European Respiratory Society (ERS) guideline on the diagnosis and management of all cases of PE clearly states that clinical presentation is non-specific in most cases, where dyspnea and chest pain can be vague. Furthermore, obesity is only listed as a weak risk factor in most cases, except for pregnant females [19]. Neither the Geneva score [5] nor the Wells score [6] includes obesity in the computation of the overall score.

The broad differential diagnosis of acute dyspnea in patients with obesity, along with their frequent and multiple co-morbidities, can complicate the diagnosis of PE. This complexity may result in missed or delayed treatment, which can have catastrophic consequences for the patient [14,18].

Upon examining the data from Shachar et al., who studied 29,610 patients with high-risk thromboembolism [4], it is noted that while 40.3% of the population in the United States is classified as obese [20], only 22.9% of patients diagnosed with high-risk PE were obese. Additionally, patients with severe obesity who had PE were significantly younger and more likely to be female [4].

While Shachar et al. suggest that obesity may have a protective effect, Table 5 indicates that another interpretation is based on estimates derived from the prevalence of obesity in the general population [20] and the HR for PE in obesity reported in the literature [9]. Table 5 shows the possibility that only between half and one third of obese patients are being diagnosed in the high-risk group, a figure similar to the 53.6% reported by Kwok et al. [11].

**Table 5:** Estimated number of undiagnosed cases using populations statistics and hazard ratio for PE in obese patients* . Estimates of missed diagnosis of PE are based on numbers reported by Shachar et al. [4]. The expected number of patients with severe PE based on the population prevalence of obesity of 40.3% [20] indicates that up to 56.0% were possibly unreported. A second additional adjustment was made by applying the hazard ratio for PE in obesity reported by Gregson et al., 1.43 for PE in patients with obesity, suggesting that 69.2% of PEs may have been unreported [9]. *Calculations are based on the assumption that all patients without obesity were diagnosed. PE: pulmonary embolism

Corrections		Patients without obesity	Patients with obesity	Total	% PE undiagnosed
Numbers reported by authors, n (%)	actual	22825 (77.1%)	6785 (22.9%)	29610 (100%)	
General population statistics, irrespective of disease, United States (Non-obese 59.7%, Obese 40.3%)*	predicted	22825	15408	38233	56.0%
Explanation: actual obese 22825 divided by 59.7 multiplied by 40.3 gives 15408 estimated number of obese patients with PE using population statistics [20]. (estimated the number of missed cases 15408 minus 6785 divided by 15408 =56.0%)		59.7%	40.3%		
Explanation: Hazard ratio for PE in obesity is 1.43/1.00 [9]. Estimated obesity cases with PE = 15408 multiplied by 1.43 = 22033. (estimated number of missed cases 22033 minus 6785, = 15248 divided by 22033 = 69.2%)	predicted	22825	22033	44858	69.2%
* Calculations are based on the assumption that all patients without obesity were diagnosed		50.9%	49.1%		

Current medical literature suggests that in patients with obesity, undiagnosed cases of PE may contribute to increased all-cause mortality rates in this population. These missed diagnoses can lead to unaccounted deaths, which may skew survival data and result in overly positive treatment outcomes for hospitalised patients with obesity affected by this condition. This distortion is likely due to selection bias, where healthier patients with obesity who have few or minor comorbidities are disproportionately represented in the survival data analysis.

The three cases reported in this report demonstrate a notable level of resilience to disease. This observation is consistent with the current hypothesis that patients with obesity may experience better outcomes because they have fat reserves that can provide an advantage during acute illness, along with a more robust cardiovascular response [3,18], but this is strictly an anecdotal observation. 

Stokes et al. explained the paradox in cardiovascular disease by two factors: firstly, “reverse causation”, where sicker patients are likely to lose weight, and “collider stratification”, where smoking-related illness is likely to result in loss of weight [21]. However, this bias can be eliminated statistically using a multivariate analysis [9]. Kalantar and Zadeh, on the other hand, insist that the paradox is real as shown by the consistency of the data even in some multivariate analysis, and state “the data is so remarkable, leaving little doubt that these observational data are beyond statistical constellations and bear biological plausibility” [22].

As summarised in the most recent ESC/ERS guideline on the diagnosis and management of acute PE, in suspected PE without hemodynamic instability, prediction scores including the revised Geneva score [5] and the Wells rule [6] are recommended [19]. D-dimer value rises in acute thrombosis due to activation of both coagulation and fibrinolysis. However, it has to be kept in mind that D-dimer has a high negative predictive value and a low positive predictive value. Therefore, D-dimer has a role in low-risk or intermediate-risk patients to rule out PE and avoid imaging and unnecessary irradiation, but not appropriate to rule out PE in high probability cases, in which case a bedside echocardiography or CTPA is recommended, especially in the presence of hemodynamic instability [19].

The Wells score [6] places particular emphasis on assigning a lower score (3 points less) when an alternative diagnosis is considered more likely than a PE. Aberegg et al. express significant criticism of this subjective decision-making process, where clinicians must determine if the likelihood of PE is greater than the combined probabilities of all other potential diagnoses. They describe this situation as another “paradox,” as it introduces a high level of subjectivity into a scoring system that should ideally be more objective and scientific [23].

In the three cases in this report, the Wells score [6] detected none of the three patients, while the Geneva score [5] successfully identified all three, albeit only as intermediate probability and not reaching the level of high probability ≥ 5 points. However, due to the small sample size, these findings should be regarded as anecdotal. Nevertheless, these cases may serve as good examples to illustrate why there is such a high rate of late or missed diagnoses observed in larger studies.

These three cases also suggest that in clinical practice, subjective assessment should be entertained with great caution in obese patients with multiple co-morbidities presenting with dyspnoea. A low threshold for performing the D-dimer test should be recommended when exercising clinical judgement. 

## Conclusions

We propose a hypothesis to explain the obesity paradox in PE. PE is often underdiagnosed, affecting a large number of patients, particularly those who have obesity with comorbidities. This misdiagnosis can lead to seemingly higher survival rates among patients with obesity due to relatively healthier individuals being identified and treated earlier, while sicker patients with multiple comorbidities may either not be diagnosed at all, or receive delayed or inadequate treatment, contributing to raised all-cause mortality for obesity.

Obesity significantly raises the risk of thromboembolism, yet it is not included in commonly used risk assessment scores for PE. Clinicians should be very vigilant when diagnosing PE in patients with obesity and maintain a low threshold for ordering D-dimer tests, especially if new respiratory symptoms arise. Studies are needed to explore the impact of missed diagnoses on treatment outcomes for PE in patients with obesity who have comorbidities and to prove or disprove this hypothesis.
